# Pelvic bone morphometric analysis in the dugong (*Dugong dugon*)

**DOI:** 10.1038/s41598-020-76545-w

**Published:** 2020-11-09

**Authors:** Korakot Nganvongpanit, Phaothep Cherdsukjai, Burin Boonsri, Kittisak Buddhachat, Patcharaporn Kaewmong, Kongkiat Kittiwattanawong

**Affiliations:** 1 Animal Bone and Joint Research Laboratory, Department of Veterinary Biosciences and Public Health, Faculty of Veterinary Medicine, Chiang Mai University, Chiang Mai, 50100 Thailand; 2 Excellence Center in Veterinary Bioscience, Chiang Mai University, Chiang Mai, 50100 Thailand; 3 Phuket Marine Biological Center, Phuket, 83000 Thailand; 4 Department of Biology, Faculty of Science, Naresuan University, Phitsanulok, 65000 Thailand

**Keywords:** Anatomy, Marine biology

## Abstract

The dugong (*Dugong dugon* Müller) is recognized as an endangered marine mammal. There is limited available anatomical data on the dugong’s skeletal system, while what is available has not been well established due to the limited number of archived samples and limited access to them. Importantly, there are certain key questions that should be answered when examining the bones and/or remains of animals such as; what kind of bone is it?; what species does it belong to?; what sex was the animal?; how old was the animal? or how big was it?, etc. In this study, we have focused on the pelvic bone of the dugong by asserting the hypothesis that pelvic bone morphology is related to age, sex, and body size. Here, we have established certain morphometric data encompassing 8 parameters and 5 indexes to analyze the morphology of the pelvic bones obtained from 88 specimens (45 dugongs). We will present three main findings: (1) the pelvic bone in mature male subjects is larger than it is in female subjects, (2) a high rate of accuracy can be established for sex identification using morphometric data obtained from the pelvic bone, and (3) the pelvic bone has the highest degree of correlation with body length, followed by body weight and age. Notably, the important data on the pelvic bone of the dugong acquired in this study can be reliable and extremely useful in sex identification and body size estimation.

## Introduction

A thorough understanding of pelvic bones (hip bone) in marine mammals, including whales, dolphins, and sirenians (manatees; *Trichechus* spp*.* and dugong; *Dugong dugon*), has not been as fully established as it has been in land mammals^1^. The pelvic bones of completely aquatic mammals have become vestigial structures that loosely resemble those of non-marine mammals. However, they do not share a full association with the vertebrae of non-marine mammals because the pelvic bones are not attached to the sacrum as firmly as they are in land mammals^2^. The major function of the pelvic apparatus in these marine animals is to serve as the muscle attachment points for the genitalia and the abdominal body wall^3–8^. Notably, the ischiocavernosus muscles insert deeply toward the distal end of the penis in male cetaceans, as the origin of these muscles is on the pelvic bone^5,9^.

Studies on the pelvic morphology and morphometric characteristics related to sex and age are well established in humans-*Homo sapiens*^10,11^ as well in various species of land mammals such as dogs *Canis familiaris*^12^, cats *Felis catus*^13,14^, hylobatids *Hylobates lar* and *Symphalangus syndactylus*^15^ and rats *Rattus norvegicus*^16^. However, in marine mammals, and in particular for an endangered species like the dugong, these data have not yet been well established. This is because bone samples for the dugong are rare and limited in number and there is limited recorded osteological data on this sea mammal. In dugongs, the pelvic bone is long and stick-like in appearance. The ilium and ischium are of subequal length and fused by the age of 5 years in both sexes^17^; whereas in manatees, the pelvic bone is more plate-like and cross-shaped in lateral view^18^. The ischium is the largest portion of the manatee pelvis, with the ilium forming a small cap on the anterior surface of the bone complex^18^.

Based on our literature review, a single publication reported on the structure of the pelvic bone of the dugong in the year 1991^17^. However, over the course of the next 29 years, no publication has reported on the morphology or morphometric data of the pelvic bone of the dugong. In general, there is limited available information on not only the pelvic bone, but also on other bones of this sea mammal. One study conducted in the year 2017 reported that the skull and scapular morphology and morphometric measurements of dugongs could be used as tools for sex identification, determination of habitat and estimation of body length^19^. Due to the fact that the dugong is an endangered species, a limited number of samples that can be collected by responsible institutes. At present, not many institutes possess a large number of dugong skeleton samples that can be used for research. This work aims to expand the existing knowledge on the morphology and morphometric data of the pelvic bone of the dugong. For this purpose, we have conducted studies involving a range of categories with regard to the dugong including age, sex, and body size. The hypothesis of our present research is that the morphology and morphometric data of the pelvis bone of the dugong are related to age, sex, and body size. The information of this study will be beneficial in the creation of a reference on dugong anatomy, biology, conservation, and forensics.

## Materials and methods

### Bone samples

Samples were obtained from the Animal Anatomy Museum, Phuket Marine Biological Center, Phuket, Thailand. A total of 88 pelvic bone samples were collected from 25 male subjects (48 bones) and 20 female subjects (40 bones). A total of 13 separate permanent tusks (from 46 dugongs) were also employed in this study to estimate the age of the subjects using the dentinal growth layer groups (GLGs) technique^20,21^. The recorded data of all animals used in this study included sex (male or female), body length, and weight.

The dry bones from dead animals used in this study did not require approval from the Animal Ethics Committee, Faculty of Veterinary Medicine, Chiang Mai University.

### Morphometric measurements

**Figure 1:**
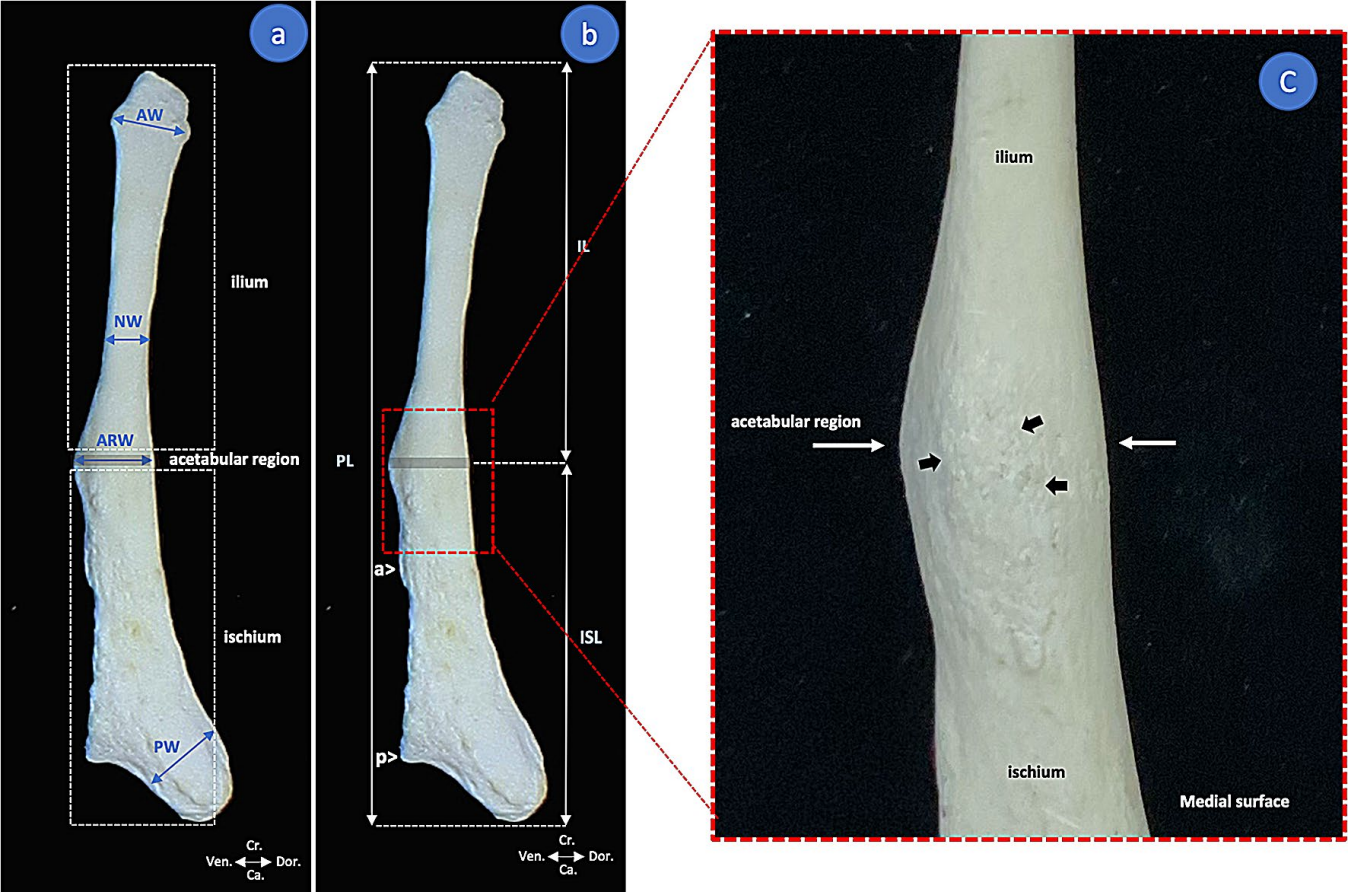
Landmarks for measurements taken from the pelvic bone of the dugong (see description of measurements in Table 1). Lateral view (**a**,**b**) of left pelvic bone (DU 040, male). The amplification photo (**c**) of the acetabular region in the medial surface found rough surface (black arrow), the landmark for the acetabular region is the maximum width of acetabular region (white arrow) (*cr*. cranial, *ca.* caudal, *dor.* dorsal, *ven.* ventral).

A total of 8 parameters were included in this study (Table 1, Fig. 1). Measurements were obtained using digital vernier calipers (Shanghai Jiuquan Hardware Tools, China) to the nearest 0.1 mm. Each measurement was recorded by a single expert who measured each object twice at intervals of 2 weeks. The results were recorded as mean values. Ultimately, the selected parameters were used to calculate 5 indexes (Table 1).

**Table 1:** Description of measurements taken from the dugong pelvic bone and the resulting indexes (see Fig. 1).

Acronym	Measurement	Description of measurement
**Parameter**		
ARW	Acetabular region width	Maximum width of acetabular region in lateral view
AW	Anterior width	Maximum width of anterior part of ilium in lateral view
CRW	Cranial width	Maximum distance between the outer margins of the zygomatic arches
IL	Ilium length	Distance from the anterior edge of the pelvis to the acetabular region
ISL	Ischium length	Distance from the acetabular region of the pelvis to the posterior-most projection of the pelvis
NW	Narrow width	Minimum width of the ilium in lateral view
PL	Pelvic length	Distance from the anterior edge of the pelvis to the posterior-most projection of the pelvis
PW	Posterior width	Maximum width of posterior part of ischium in lateral view
**Index**		
AW/PW	Anterior width/posterior width	
BL/PL	Body length/pelvic length	
IL/ISL	Ilium length/ischium length	
PL/IL	Pelvic length/ilium length	
PL/ISL	Pelvic length/ischium length

### Measurements of dentinal growth layer groups to estimate age

**Figure 2:**
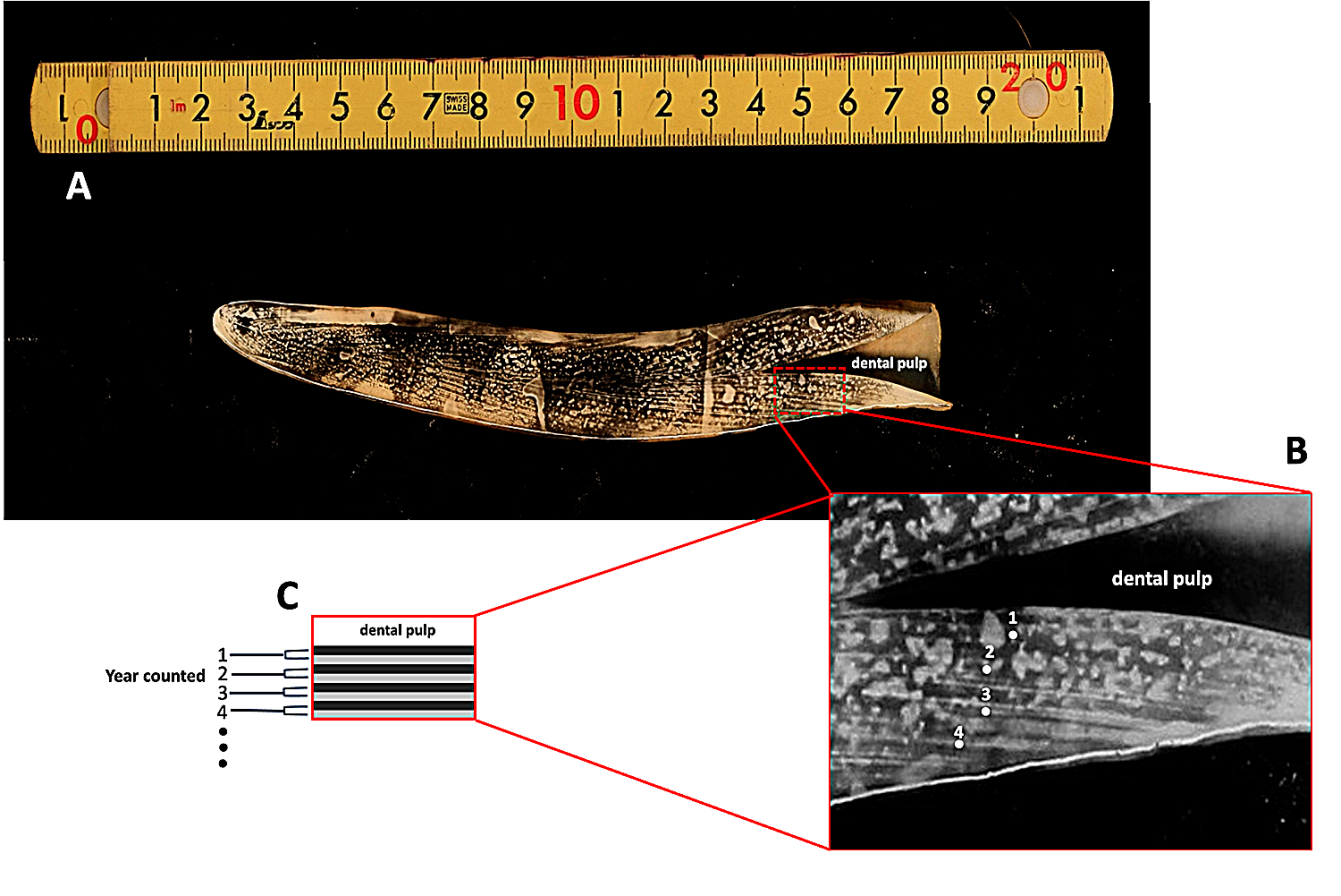
A representative photo of counted dentine growth layer groups in the tusk of a 24-year-old female dugong (**A**). Each GLG is represented (amplification photo shown in (**B**)) by one light and one dark band, counted from the base of the dental pulp to the tip of the tusk (**C**).

The counting of the dentinal GLGs followed previously established protocols^20,21^. The tusks were bisected longitudinally in the mid-sagittal plane using a Buehler Isomet low speed saw (Struers Minitom, Denmark), polished using sandpaper, and then etched for 1–3 h. in 10% formic acid. Tusks were then washed in water for 10 min to remove the etching reagent, followed by 2–3 min in 100% acetone to increase the clarity of the GLGs. Samples were washed again in water overnight to remove the formic acid. The etched tusks were then dried at room temperature and then rubbed with laser toner powder (Canon, Thailand). The number of GLGs present in each tusk was counted in triplicate (Fig. 2).

### Study design and statistical analysis

#### Descriptive morphology

Fused and non-fused associations between the ilium and ischium were observed. The morphology of the pelvic bone between male and female dugong subjects was observed in order to seek a hallmark that could be used for sex identification.

#### Correlation between biological data (age, body length and body weight) and morphometric data

The age of dugongs acquired from the GLGs was used to study the correlation between age and the morphometric data (parameter and index). The body length and body weight of all dugongs were used to establish a correlation with the morphometric data.

A linear regression model involving the biological data and morphometric data was analyzed using R version 3.6.3^22^ to establish the best equation for age prediction.

#### Sex determination using pelvic morphometric equations

A dugong was categorized as mature when the body length was over 2250 mm and a dugong was categorized as immature when the body length was measured at 2250 mm or lower. These categorizations were based on published data from northern Australia which established that minimum length of mature dugongs of either sex can range from 2200 to 2500 mm^23^.

Before employing the Principal Component Analysis (PCA), we compared the differences between male and female subjects in 3 separate body length groups; G1 (body length 1500–1999 mm), G2 (body length 2000–2499 mm) and G3 (body length 2500–3000 mm) using either a t-test for normally distributed parameters or the Mann–Whitney U test for non-normally distributed parameters. Notably, P-values of < 0.05 were considered statistically significant. The PCA was then performed for sex clustering and the data were analyzed in three different categories; combined (immature + mature), immature and mature using the parameter index, and mixed (parameter + index).

## Results

### Descriptive morphology

**Figure 3:**
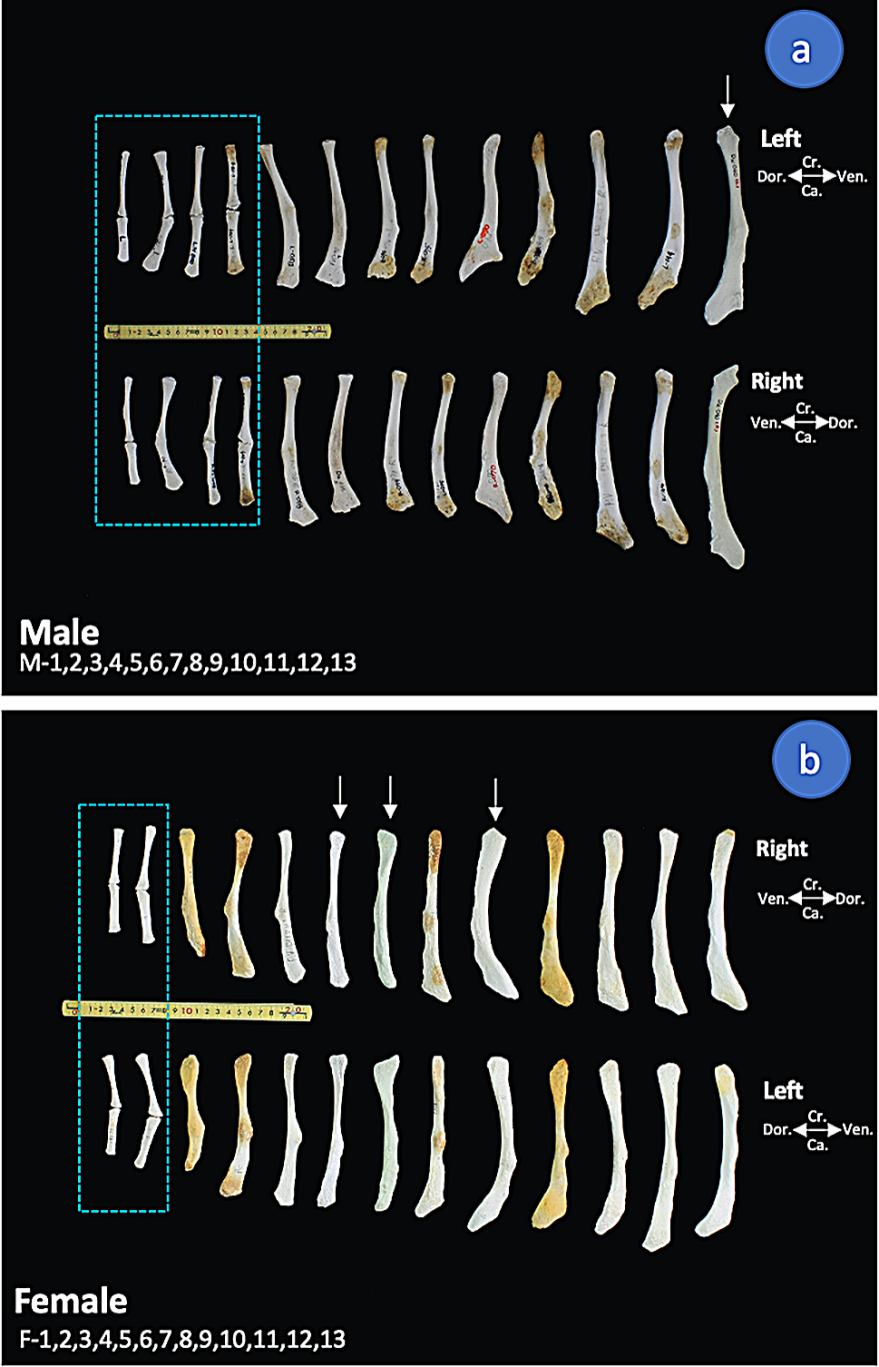
Representatives of morphometric variations in dugong pelvic bones, medial views. Non-fused pelvic bones (blue rectangles) were found in all juvenile dugong subjects and some immature dugong subjects. Some dugong subjects displayed slight differences in the morphology of the pelvic bones between the right and left sides (white arrows).The bones of the male dugongs shown include: Du 140 (M1), Du 130 (M2), Du 047 (M3), Du 084 (M4), Du 058 (M5), Du 038 (M6), Du 074 (M7), Du 075 (M8), Du 070 (M9), Du 088 (M10), Du 243 (M11), Du 144 (M12), and Du 40 (M13). The bones of the female dugongs shown include: Du 260 (F1), Du 126 (F2), Du 057 (F3), Du 129 (F4), Du 241 (F5), Du 016 (F6), Du 036 (F7), Du 234 (F8), Du 078 (F9), Du 048 (F10), Du 291 (F11), Du 233 (F12), and Du 292 (F13). (*cra*. cranial, *cau.* caudal, *dor.* dorsal, *ven.* ventral).

A total of 23 specimens of 25 immature dugong subjects (body lengths lower than 2250 mm) had non-fused pelvic bones (Fig. 3). In the group of subjects for which we had determined their age (26 bones) by GLG technique, we found non-fused pelvic bones in subjects that were 8 and 6 years old, while those with an age of over 14 years had fused pelvic bones. In the group of subjects with unknown age, 44 bones were obtained from 25 male dugong subjects and 40 bones were obtained from 20 female dugong subjects. Among males of body length 2210 mm and lower, 28 had non-fused pelvic bones and 4 had fused pelvic bones. Of males of body length 2259 mm and higher, all 16 had fused pelvic bones. Among females of body length 2000 mm and lower, all 18 had non-fused pelvic bones, while those with body length of 2250 mm and over, all 22 subjects had fused pelvic bones (Table 2).

**Table 2:** Fusion of ilium and ischium of dugong pelvic bone (n = number of pelvic bones).

Pelvic fusion	By age (n = 26)	By body length	
		Male (n = 44)	Female (n = 40)
Non-fused	8 years and lower (n = 4)	2210 mm and lower (n = 28*)	2000 mm and lower (n = 18)
Fused	24 years and higher (n = 22)	2259 mm and higher (n = 16)	2250 and higher (n = 22)

*4 additional samples (obtained from 2 dugong subjects having body lengths lower than 2210 mm) also presented fused pelvic bones.

**Figure 4:**
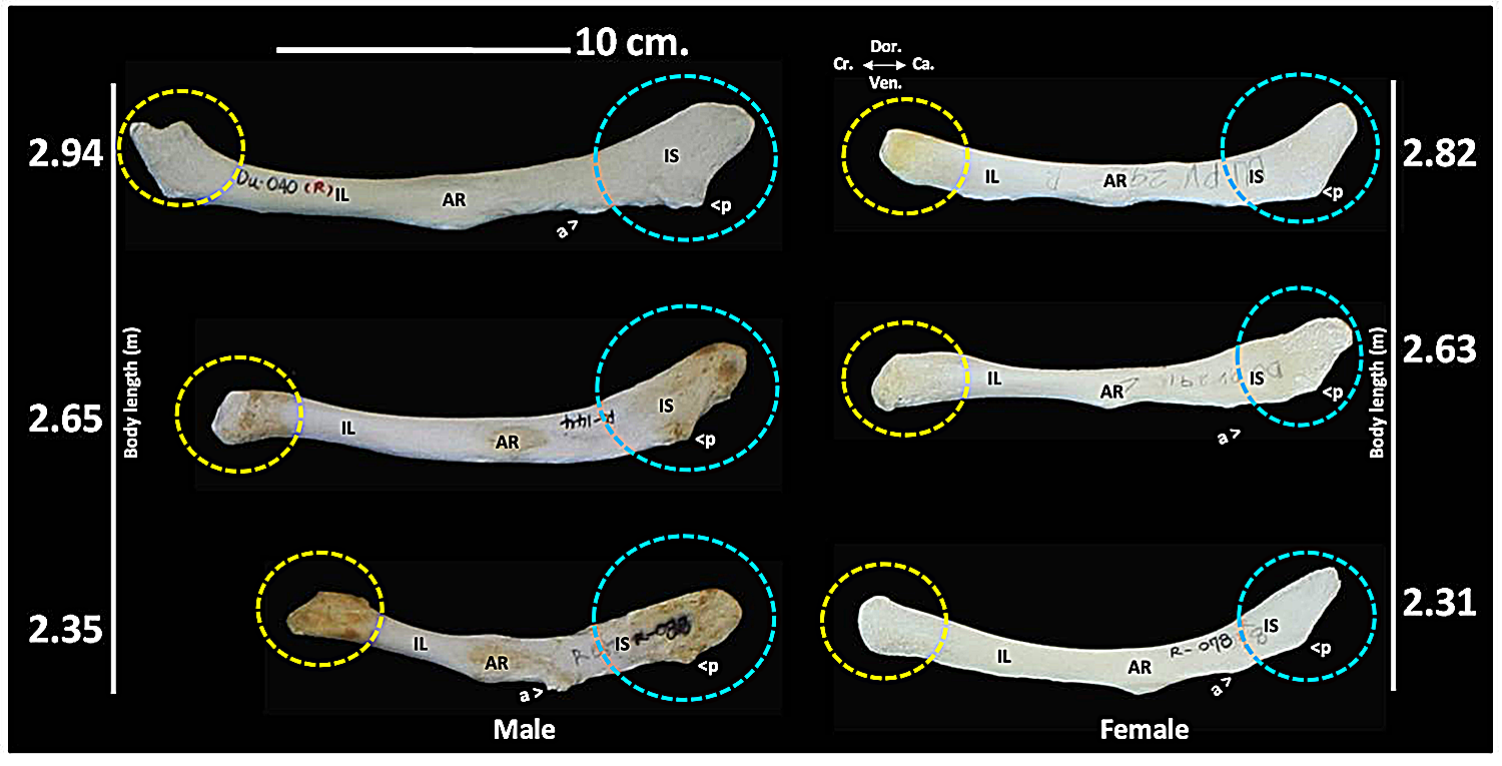
Representation of morphometric variations in the right pelvic bone for male and female dugong subjects of similar body length. In medial view, there are variations of the anterior end (yellow circles) and posterior end (blue circles) of the pelvic bones. The presence of an anterior ventral process of the ischium (a) and a posterior ventral process of the ischium (p) were observed in some specimens. DU 040 (male, 2.94 m), Du 144 (male, 2.65 m), DU 088 (male, 2.35 m), DU 292 (female, 2.82 m), DU 291 (female, 2.63 m), and DU 078 (female, 2.31 m). (*AR* acetabular region, *cra.* cranial, *cau.* caudal, *dor.* dorsal, *IL* ilium, *IS* ischium, *ven.* ventral).

Variations in pelvic bone morphology were observed in each sex, but no specific hallmark was established for sex identification (Fig. 3). Even in the same dugong, slight differences between the right and left pelvic bones were observed (Fig. 3, white arrows). In a comparison between male and female subjects of similar body sizes, a significant hallmark was not found, even though some differences were observed in the morphology of the anterior ends (Fig. 4, yellow circles) and the posterior ends (Fig. 4, blue circles) of the pelvic bone. Additionally, the presence or absence of an anterior ventral process, along with the sizes of the anterior and posterior ventral processes of the ischium, were not found to be sex-specific.

### Correlation between biological data (age, body length and body weight) and morphometric data

**Figure 5:**
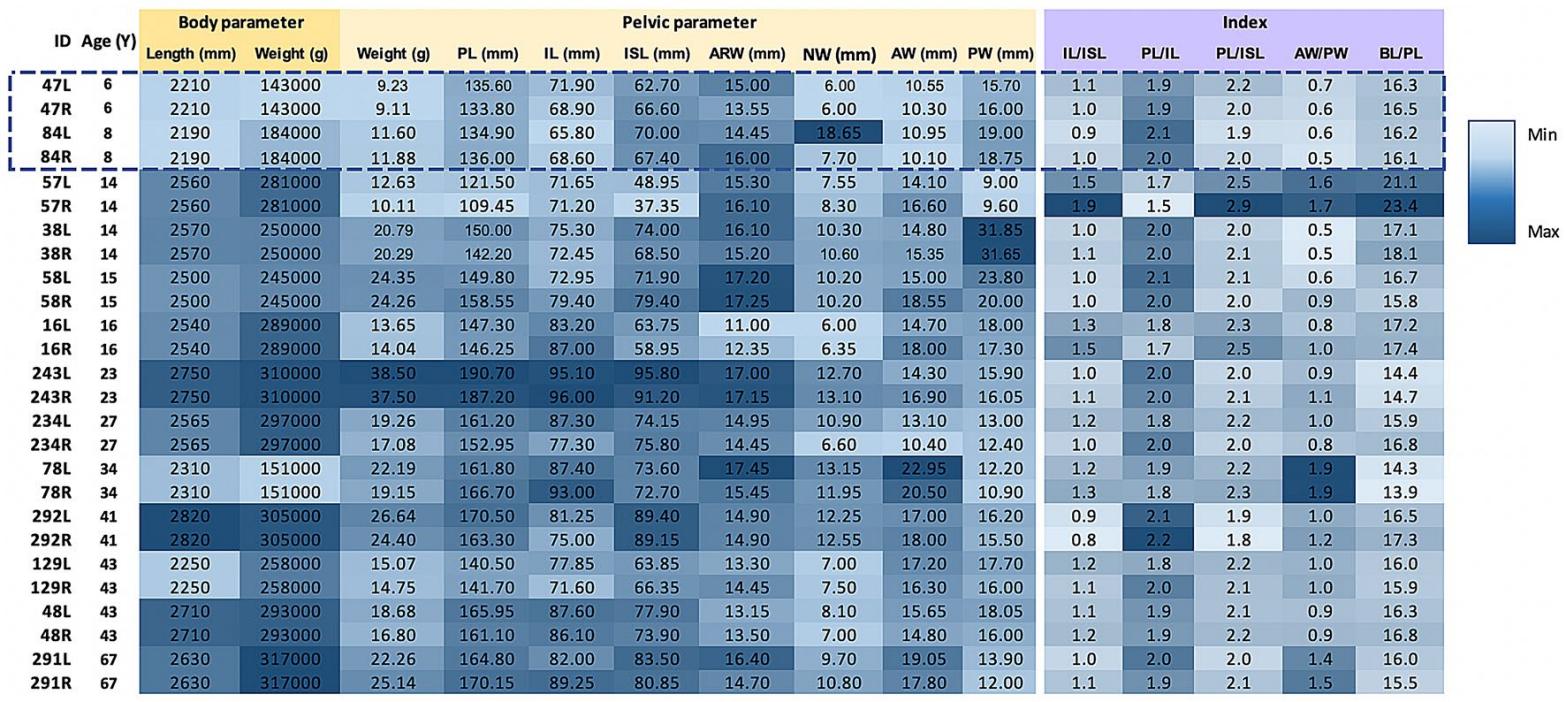
Heat map generated from the values of each parameter and index for 26 pelvic bones (13 dugongs). A rectangular blue dotted box indicates non-fused pelvic bones.

A total of 26 dry pelvic bones of dugong subjects, including 10 males (4 subadults) and 16 females whose ages were established through GLG analysis, were used in the age prediction study. Ages ranged from 6 to 67 years old with a mean ± SD value = 27 ± 17.38. All values of pelvic parameters and indexes are presented in a heat map (Fig. 5). Of all parameters, seven regression models were established from a stepwise AIC regression analysis (Table 3). A diagnostic analysis of each model was performed to check for normality, heteroscedasticity, and influential observations. Raw and mixed models of the male group were found to be the best models in terms of age prediction, with a high adjusted R square and the lowest residual standard error among all of the models. However, the index model of the male group was not found to be good enough for age prediction due to the insignificance of the P-value. The models pertaining to the pooled group and the female group were also poor in terms of age prediction due to low adjusted R square and high residual standard error, although a P-value of significance was still applied.

**Table 3:** Linear equations for age, body length, and body weight prediction derived from stepwise linear regression analysis.

Model	Function	Adj. R^2^	P-value	RSE
**Age prediction**				
Combined (male + female)				
Parameter^#^	Y = 2.55(A) + 0.46(ISL) − 46	0.35	0.002	13.94
Index	Y = 45.474 + 30.99(A/P) − 44.1(IL/ISL)	0.39	0.001	13.59
Mixed^#^	Y = 2.55(A) + 0.46(ISL) − 46	0.35	0.002	13.94
Male				
Parameter	Y = 0.3(PL) + 0.27(P) − 38.36	0.97	0.000	1.04
Index	Y = 22.17 + 20.31(A/P) − 22.36(IL/ISL)	0.30	0.118	5.26
Mixed	Y = 41.35(PL/IL) + 2.26(BL/PL) + 0.72(IL) − 160.9	0.99	0.000	0.65
Female				
Parameter*	Y = 0.82(ISL) − 22.46	0.44	0.003	12.36
Index	Y = 86.46 − 42.27(ISL/IL)	0.39	0.005	12.93
Mixed*	Y = 0.82(ISL) − 22.46	0.44	0.003	12.36
**Body length prediction**				
Combined (male + female)				
Parameter	Y = 682.9 + 11.32(PL)	0.84	0.000	190.8
Index	Y = 1892.95(PL/IL) + 977.18(PL/ISL) + 546.98(A/P) − 79.51(BL/PL) − 2686.46	0.36	0.000	373.6
Mixed	Y = 19.17(IL) + 12.19(ISL) + 69.3(BL/PL) − 1104.89	0.93	0.000	127.5
Male				
Parameter	Y = 493.95 + 8.09(IL) + 77.2(ARW)	0.87	0.000	151.2
Index	Y = 1804.64 + 674.96(PL/IL) − 59.1(BL/PL)	0.19	0.003	370.32
Mixed	Y = 16.94(IL) + 12.51(ISL) + 9.89(P) + 83.87(BL/PL) − 10.21(A) − 1260.51	0.97	0.000	68.8
Female				
Parameter	Y = 598.92 + 12.42(PL)	0.87	0.000	191.3
Index	Y = 4143.68(PL/IL) + 2723.85(PL/ISL) + 436.14(A/P) − 117.53(BL/PL) − 9905.01	0.58	0.000	360.9
Mixed	Y = 15.75(PL) + 55.4(ARW) + 67.24(BL/PL) − 42.27(NW) − 1416.43	0.95	0.000	120.7
**Body weight prediction**				
Combined (male + female)				
Parameter	Y = 2043.3(PL) − 79,613.2	0.76	0.000	42,870
Index	Y = 468,868(PL/IL) + 267,864(PL/ISL) + 80,557(A/P) − 14,131(BL/PL) − 1,115,231	0.34	0.012	71,060
Mixed	Y = 2876.7(PL) + 12,941.3(BL/PL) − 410,792.4	0.85	0.000	33,870
Male				
Parameter	Y = 2068.2(PL) − 92,844.5	0.85	0.000	28,240
Index	Y = 50,078 + 167,176(PL/IL) − 12,024(BL/PL)	0.29	0.000	62,480
Mixed	Y = 2482.4(PL) + 1760.2(P) + 11,366.7(BL/PL) − 366,168.7	0.94	0.000	17,680
Female				
Parameter	Y = 2043.2(PL) − 68,462.5	0.72	0.000	53,290
Index	Y = 833,125(PL/IL) + 608,648(PL/ISL) −22,079(BL/PL) − 2,303,445	0.39	0.000	78,340
Mixed	Y = 3845.4(IL) + 2155.6(ISL) + 14,191.7(BL/PL) − 452,235	0.79	0.000	45,250

*Y* predictor; age (years), body length (mm) or body weight (g), *Adj.R*^*2*^ adjusted R-square, *RMSE* root mean square error, *Parameter* using raw value of each parameter in calculation, Index = using Index value in calculation, *Mixed* using parameter and index value in calculation.

Same symbol (*, ^#^) had a similar, function, adjust R^2^, P-value and RSE.

The model used for body length prediction revealed a high correlation (R^2^ > 0.80, P < 0.05) in male, female, and combined sexes using a designated parameter and the mixed parameters of the index (Table 3). However, in terms of a body weight prediction, a high correlation (R^2^ > 0.80, P < 0.05) was observed in male subjects when both a designated parameter and the mixed parameters of the index were used. Additionally, the combined sexes used the mixed parameters of the index. However, no high correlation (R^2^ > 0.80) was observed in the female subjects (Table 3).

### Sex determination using pelvic morphometric equations

**Figure 6:**
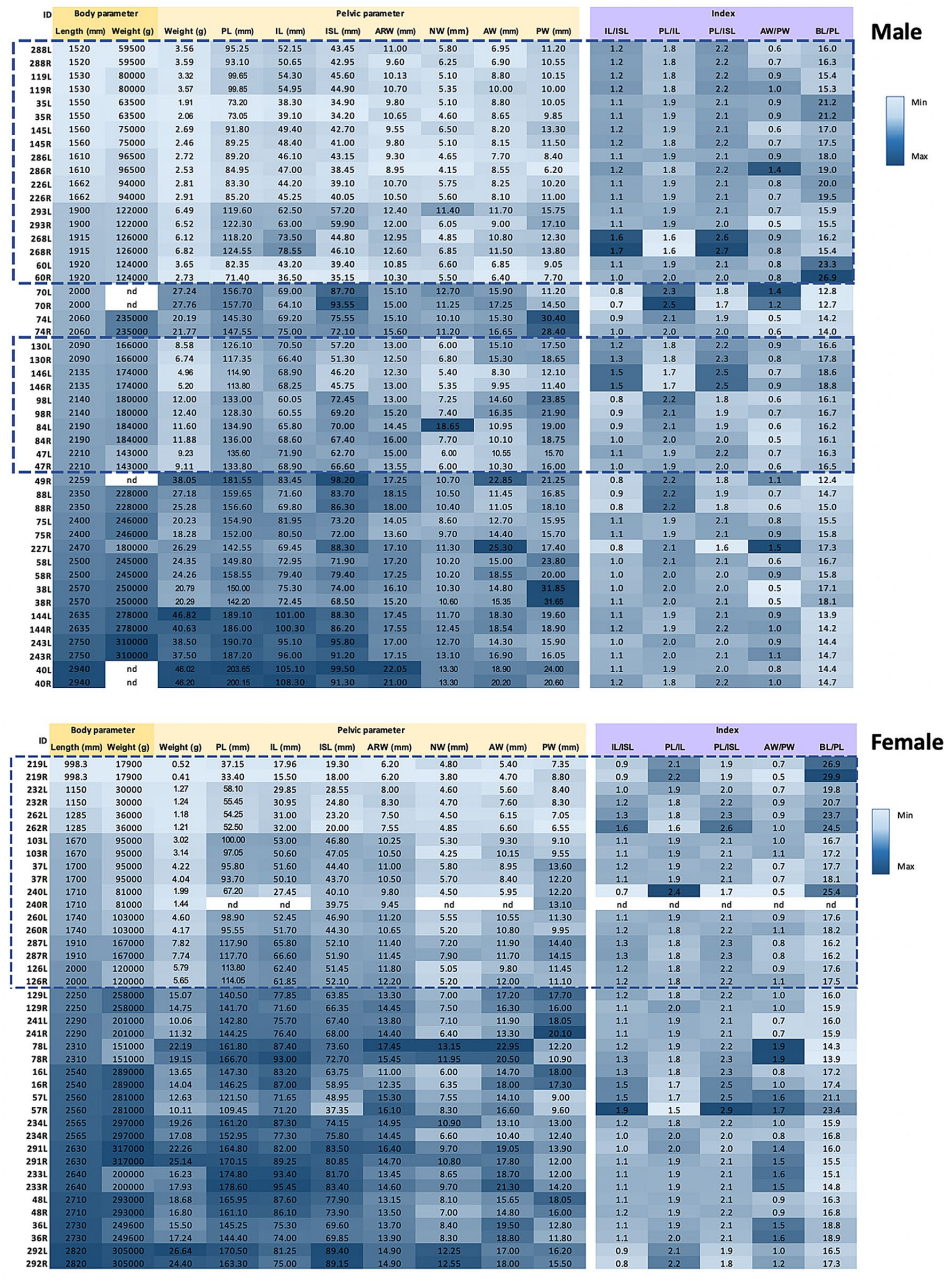
Heat map generated from the value of each parameter and index of 48 pelvic bones of 25 male dugong subjects and 40 pelvic bones of 20 female dugong subjects. A rectangular blue dotted box indicates non-fused pelvic bones (*nd* not determined).

**Figure 7:**
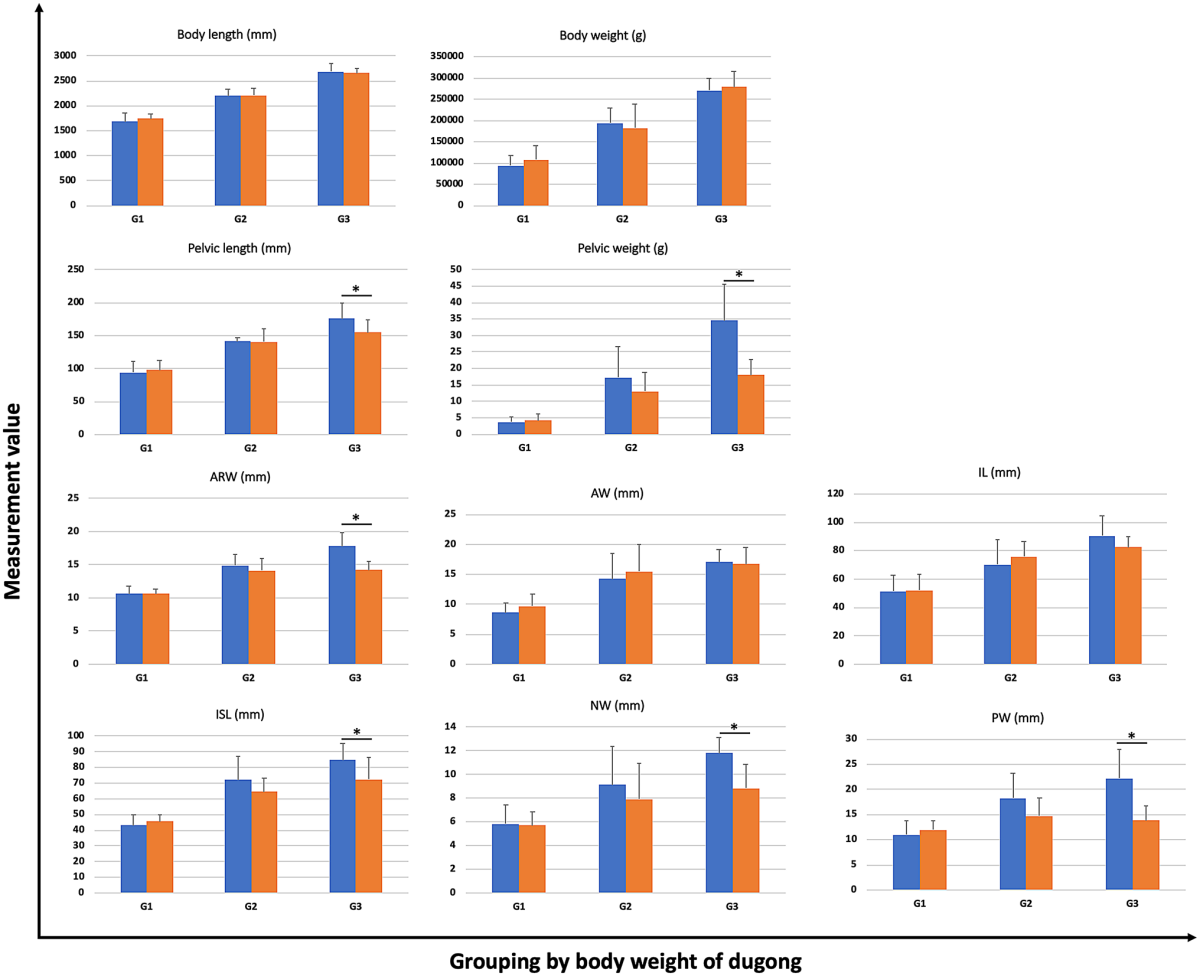
Comparative analysis between male and female dugong subjects using 10 parameters. Dugong subjects were categorized into 3 groups according to body length; G1: 1500–1999 mm, G2: 2000–2499 mm, and G3: 2500–3000 mm. The symbol * indicates significant differences between male (blue) and female (orange) subjects.

All values of the pelvic parameters and indexes in male and female dugong subjects are presented as a heat map (Fig. 6). Specifically, 10 parameters between male and female dugong subjects were compared among the 3 different body length groups; G1 (body length 1500–1999 mm), G2 (body length 2000–2499 mm) and G3 (body length 2500–3000 mm) It was found that 6 parameters, including pelvic length, body weight, ARW, ISI, NW, and PW, were significantly higher (P < 0.05) in male dugong subjects than in female dugong subjects (Fig. 7).

**Figure 8:**
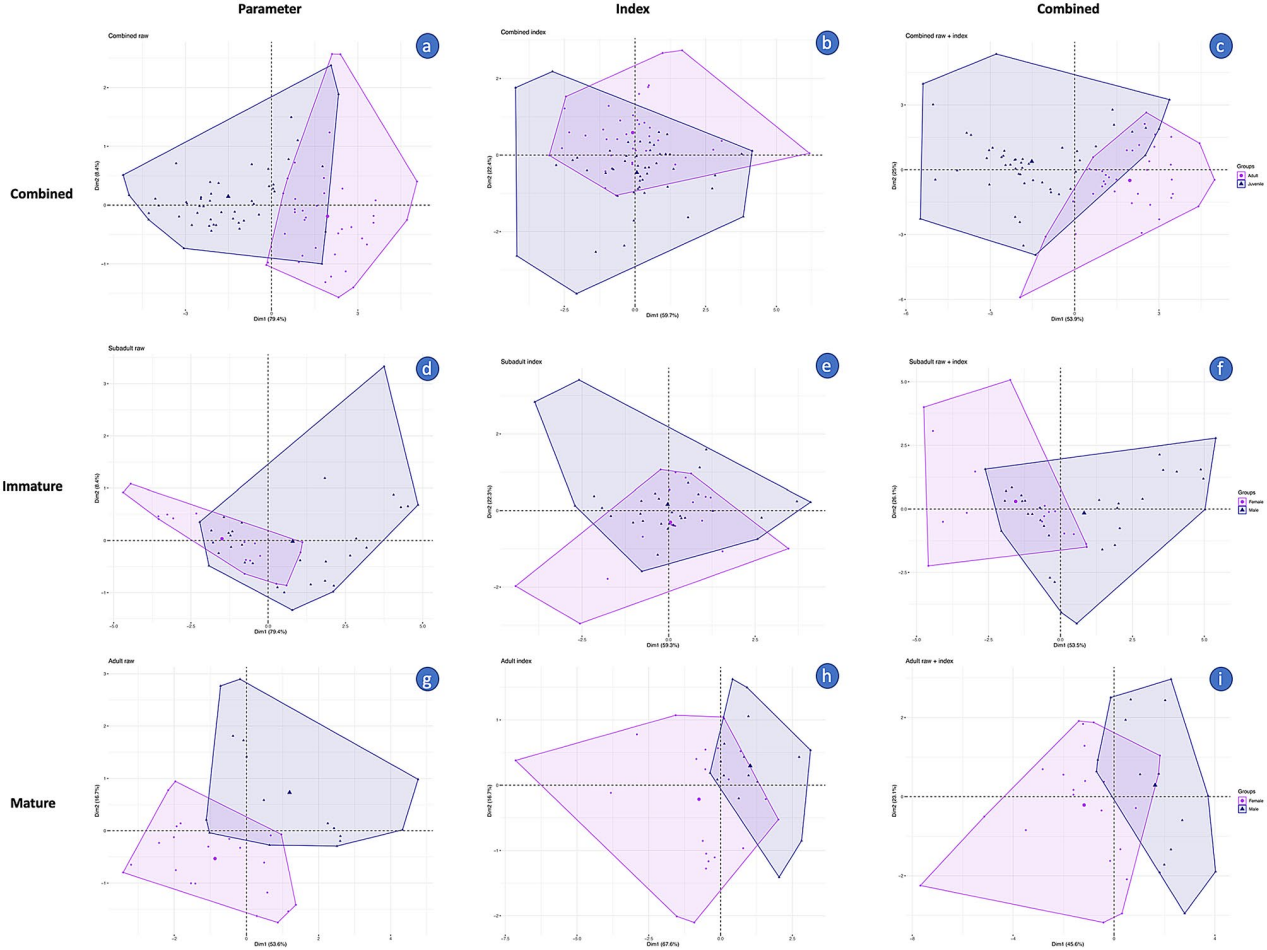
Principal component analysis for sex determination acquired from variations in the data set. A comparison of data was made between immature and mature dugong subjects, analyzed with only parameter (**a**), only index (**b**), and combined parameter and index (**c**). Data of immature dugong subjects were analyzed using only parameter (**d**), only index (**e**) and combined parameter and index (**f**). Data of mature dugong subjects were collected and analyzed with only parameter (**g**), only index (**h**) and combined parameter and index (**i**). Male subjects are indicated by blue color and female subjects are indicated by pink.

The visualized individual plot of PCA shows a separation between the groups (Fig. 8). To support this separation, the multivariate analysis of variance (PERMANOVA) was performed to validate the differences between the groups using the Euclidean distance measurement (Table 4). Individual plots using raw and index values showed a clear separation (P < 0.05) between life stages (between immature and mature stages), as well as between the immature and the mature group (between male and female subjects). However, the PCA plot of the comparison between sexes in the immature group displayed no differences when using only index values in the analysis.

**Table 4:** P-value derived from PERMANOVA.

Category of test	Combined	Raw	Index
**Life stage**			
Immature vs mature	0.001	0.001	0.01
**Immature**			
Male vs female	0.002	0.002	0.2
**Mature**			
Male vs female	0.001	0.001	0.003

Stepwise logistic regression was applied to the selected parameters, which generated five models for sex prediction at each life stage (Table 5). In the mature group, the logistic function displayed a high degree of accuracy at up to 92.1% and 84.21% in sex estimation using both the raw model and index model, respectively. However, the accuracy of sex estimation in the mixed model of the mature subjects was tapered to 76.31%. In the immature group, no model of the logistic function was good enough for the classification of sex, with degrees of accuracy in the range of only 67.37–69.38%.

**Table 5:** Logistic function for sex determination derived from stepwise logistic regression analysis.

Model	Function	Accuracy (%)	Precision (%)
**Combined (immature + mature)**			
Parameter	Y = 3.95 + 0.39(A) – 0.5(ARW) – 0.18(P)	74.71	74.91
Index	Y = 3.55(A/P) + 0.25(BL/PL) – 7.82	71.26	64.87
Mixed	Y = 0.11(IL) + 0.39(A) + 0.36(BL/PL) – 0.95(ARW) – 0.17(P) – 4.18	77.01	74.40
**Immature**			
Parameter	Y = 7.73 + 0.35(A) − 1.08(ARW)	69.38	67.18
Index	Y = 0.23(BL/PL) − 5.07	67.34	58.45
Mixed	Y = 7.73 + 0.35(A) − 1.08(ARW)	69.38	67.18
**Mature**			
*Parameter*	*Y* = *37.11* + *0.48(PL)* + *0.53(A) *−* 0.62(IL) *−* 0.52(ISL) *−* 1.26(ARW) *−* 0.56(P)*	*92.10*	*91.47*
*Index*	*Y* = *5.03(A/P)* + *1.38(BL/PL) *−* 10.82(PL/IL) *−* 5.61*	*84.21*	*83.08*
Mixed	Y = 0.07(PL) + 0.47(BL/PL) − 0.13(ISL) − 0.45(NW) − 4.84	76.31	75.28

The italics value indicate logistic functions with good ability for classification.

Probability = 1/(1 + e^(-y)^); male < 0.5; female > 0.5.

Parameter = using raw values of parameters in calculation, Index = using Index value in calculation, Mixed = using parameter and index values in the calculation.

## Discussion

The highlights of this study reveal a high rate of accuracy of up to 92.10% for sex identification using the parameters of the pelvic bones of mature dugongs. Moreover, the pelvic bones in mature male dugongs were larger than in the female dugongs. Additionally, we identified a high degree of correlation between age, body length, and body weight by using the established parameters and indexes of the pelvic bones.

### Morphology of pelvic bones

Our study found that the ilium and ischium were not fused in the group of subjects aged 6 and 8 years old, while a fused pelvic bone was observed in subjects at ages of 14 years old and over. Therefore, we assume that pelvic bone fusion occurred between 9 and 13 years of age. However, our study could not indicate the exact age of fusion because we did not have access to samples in all age ranges. A previous study reported that fusion of the ilium and ischium occurred when the dugong was around 5–8 years old, and the pelvic bone was completely fused (with the suture obliterated) when the dugong was older than 8 years^17^. We likewise found that a fused ilium and ischium occurred in dugongs that were 5 to 8 years old. This was the case with dugongs that were living in the sea of Thailand and the findings were similar to the specifications of dugongs living in the sea of Australia. A previous study reported that mature dugongs had a body length ranging between 2200 and 2500 mm^23^. However, our study found that a fused pelvic bone was observed when the body size was over 2250 mm in both sexes. Thus, this figure may indicate the maturation size of dugongs even among those living in widely different locations (the sea of Thailand and the sea of Australia).

However, we could not find any significant hallmark for the pelvic bone that could be used for sex identification. Previous studies involving the scapula of the dugong reported a hallmark for sex identification by scapular morphology using the caudal border tubercle and coracoid process at 91.30% and 96.15% accuracy rates for identifying males and females^19^. The morphology of the dugong pelvic bone indicated a high variation in male and female subjects. In other species, such as humans, variations of the pelvic bone according to sex were observed^24^. However, similar significant differences in the dugong were not observed. A key for identifying the sex of dugong pelvises was proposed in 1991^17^; however, we did not have success when applying this key because some criteria were considered too subjective. Importantly, significant hallmarks are the key to successful identification of gender. In other marine mammals, significant hallmarks in the pelvic bone could be used for sex identification. For example, in a study involving North Pacific common minke whales (*Balaenoptera acutorostrata*), the shape of the pelvic bones clearly differed depending on sex^25^. Specifically, the pelvic bones of adult females were flat, while those of adult males consisted of two types: having a twisted caudal portion or a thickened caudal portion.

### Correlation between biological data and pelvic bones

Morphometric analysis of the pelvic bone revealed a significantly high correlation between age and the morphometric data in male subjects (R^2^ = 0.99, P < 0.01), but a low correlation in female subjects (R^2^ = 0.44, P < 0.01). However, this determination might have been influenced by the number of samples applied in this study; male dugongs comprised 10 pelvic bones (from 5 dugongs) whereas female dugongs comprised 16 pelvic bones (from 8 dugongs). When male and female subjects were combined, a low degree of correlation was observed with any degree of significance (R^2^ = 0.35–0.39, P < 0.01) between age and the morphometric data. However, we were not able to increase the number of samples even though we had access to a total number of 88 pelvic bone samples taken from 25 male subjects (48 bones) and 20 female subjects (40 bones). The ages of the dugongs included in this study were established based on the GLGs counting technique. In this study we had access to only 13 dugongs with complete tusks that could be used to estimate age. Previous studies found that some parameters were related to age, e.g. the dental growth layers in the group of animals with tusks^20,21^, body length^26^ and those with skull sutures^27^.

Remarkably, we found that the pelvic bone displayed a high correlation with body length and body weight in both male and female dugong subjects. Furthermore, it was found that even though the pelvic bone in marine mammals may display poor development, the size of the bones in other parts of the body could still be used to indicate the size of the subject. This outcome would further indicate that bone size is in fact related to body size. A previous study involving 130 individual pelvic bones of 29 species of cetaceans found that pelvic bone size had a high correlation with the testes mass in male subjects^28^. Additionally, in a study involving bottlenose dolphins (*Tursiops truncatus*), it was found that pelvic bone length was corelated with body length^29^. Our study likewise found a high correlation between pelvic bone size and body size in dugongs. Moreover, the pelvic bones in mature male dugongs tended to be larger than in female dugongs. Similarly to other marine mammals, such as bottlenose dolphins, we found that the pelvic bones of males dugongs were significantly longer, heavier, and more robust than those of females^29^.

The pelvic bones of immature dugongs provided a lower rate of accuracy for sex prediction than did the pelvic bones of mature dugongs. This was because in mature subjects the bones did not show any changes in morphology^30^. Our study used 6 parameters of the pelvic bone to achieve the highest rate of accuracy for sex identification at up to 92.10%. A previous study involving other bones of the dugong reported an accuracy rate of up to 96.7% for the skull, 81.8% for the cranium, 78.4% for the mandible, and 68.1% for the scapula^19^. A study involving bottlenose dolphins using morphometric data acquired from pelvic bones reported a high accuracy rate of up to 97%^29^. Furthermore, in a human study, the pelvic bone provided an accuracy rate for sex identification of up to 97.5%^31^. Taken together, the outcomes of this study and those of previous studies affirm that the best bone that can be used for sex identification in dugongs is the skull (cranium + mandible), followed by the pelvic bone, the cranium, the mandible, and the scapula.

In field use when some unknown samples were obtained, it was determined that we would use the equation presented in Table 3 to estimate age, body length and body weight of the subjects. Moreover, sex prediction could be achieved by using the appropriate equation presented in Table 5. However, there are some limitations to each condition that we had previously described. Among the established equations, it was determined that the following equation, “Y = 37.11 + 0.48(PL) + 0.53(A) − 0.62(IL) − 0.52(ISL) − 1.26(ARW) − 0.56(P)”, should be used for the sex determination of adult pelvic bones. In terms of the age prediction of male subjects, the equation “Y = 41.35(PL/IL) + 2.26(BL/PL) + 0.72(IL) − 160.9” was used, but a low correlation was observed among female subjects when applying this equation. In terms of body length and body weight predictions, equations were selected using only identified parameters or a combination of parameters and the index which resulted in a similar prediction rate.

### Limitations of the study

It is very difficult to design a standard experiment involving this sea mammal for a number of reasons, such as the limited number of specimens available. Additionally, the recorded data must be considered incomplete because most samples were obtained from stranded dugongs. As the age of these animals is still unclear, we have to use other techniques to estimate age. Factors influencing this outcome include the low number of dugongs we had access to in this study whose ages were known. For these reasons, we believe that all studies conducted on dugongs would have value, even small-sized studies. Consequently, in order to improve the existing knowledge of dugong anatomy and biology, scientists and researchers will need to merge the findings of various studies.

## Conclusion

The study of the morphology and acquired morphometric data on the pelvic and other bones of the dugong can provide useful information on the anatomy, biology, and conservation of this sea mammal. The findings of this study can also provide forensic benefits to those conducting further research on the dugong. From this study, we have established that the pelvic bone of the dugong varies according to sex, as the pelvic bone was observed to be larger in males than in females. The findings of the morphological study indicated a correlation between age and body size. Finally, the morphometric data did provide a high accuracy rate for sex identification of the dugong.
